# Causes for Emergency Hospitalization of Neurological Patients With Palliative Care Needs

**DOI:** 10.3389/fneur.2021.674114

**Published:** 2021-08-02

**Authors:** Anna-Christin Willert, Christoph J. Ploner, Alexander B. Kowski

**Affiliations:** Department of Neurology, Charité—Universitätsmedizin Berlin, Berlin, Germany

**Keywords:** emergency hospitalization, palliative care needs, neuropalliative care, emergency department, palliative care, reason for admission, neurological patients

## Abstract

**Background:** Acute and unexpected hospitalization can cause serious distress, particularly in patients with palliative care needs. Nevertheless, the majority of neurological inpatients receiving palliative care are admitted *via* an emergency department.

**Objective:** Identification of potentially avoidable causes leading to acute hospitalization of patients with neurological disorders or neurological symptoms requiring palliative care.

**Methods:** Retrospective analysis of medical records of all patients who were admitted *via* the emergency department and received palliative care in a neurological ward later on (*n* = 130).

**Results:** The main reasons for acute admission were epileptic seizures (22%), gait disorders (22%), disturbance of consciousness (20%), pain (17%), nutritional problems (17%), or paresis (14%). Possible therapy limitations, (non)existence of a patient decree, or healthcare proxy was documented in only 31%. Primary diagnoses were neoplastic (49%), neurodegenerative (30%), or cerebrovascular (18%) diseases. Fifty-nine percent were directly admitted to a neurological ward; 25% needed intensive care. On average, it took 24 h until the palliative care team was involved. In contrast to initially documented problems, key challenges identified by palliative care assessment were psychosocial problems. For 40% of all cases, a specialized palliative care could be organized.

**Conclusion:** Admissions were mainly triggered by acute events. Documentation of the palliative situation and treatment limitations may help to prevent unnecessary hospitalization. Although patients present with a complex symptom burden, emergency department assessment is not able to fully address multidimensionality, especially concerning psychosocial problems. Prospective investigations should develop short screening tools to identify palliative care needs of neurological patients already in the emergency department.

## Introduction

After cancer, neurologic conditions are the second most common diagnosis of inpatients receiving specialist palliative care (1, 2). Like the majority of inpatients with other lifetime-reducing diseases and palliative care needs, patients with neurological diseases or complications are predominantly admitted to hospital *via* an emergency department (ED) (3, 4). Acute admission to the ED can cause serious distress in this vulnerable group due to long waiting times, lack of appropriate communication, and insufficient control of symptoms (5). However, ED visits increase with impairment and with decreasing lifetime (6–8). In many cases, they ultimately lead to long hospitalization (7, 9).

An early integration of palliative principles in the trajectory of hospital care is therefore an important aim (10).

Supplementary to disease management in the primary treating department, hospital-based specialist palliative care can be incorporated in the care of patients with life-limiting diseases by consultations of a multi-professional palliative care service. A physician and nurse both specialized in palliative care work alongside and in collaboration with the attending physician. They aim to address symptom management, help to define goals-of-care according to the (alleged) patient will, support patients with advance care planning, and provide psychosocial help for informal caregivers. The multi-professional team approach also includes additional support from social workers, psychologists, physiotherapists, and occupational and speech therapists and pastoral care. To establish a palliative care treatment plan, during the first specialist palliative care consultation an assessment is performed, which evaluates unmet palliative care needs on the physical, social, psychological, and spiritual level.

Specialist palliative care consultation is able to improve symptom burden, patient's and caregiver's satisfaction, and quality of life, and it reduces length of stay and overall healthcare costs (11, 12). Early specialist palliative care consultation is also associated with a lower in-hospital mortality rate compared with late initiation (13). In order to move palliative care “upstream” in the trajectory of in-hospital care, an implementation of palliative care in the ED has been proposed (10). Methods to achieve this goal range from education of emergency physicians in palliative care principles encouraging them as primary providers to implementation of specialist palliative care consultation by a multi-professional palliative care service as secondary providers in the ED (10, 14).

Increasing awareness for palliative care needs in the ED may allow for an early integration of palliative care in hospital. Studies throughout the past decade have mainly concentrated on ED visits of patients with end-stage cancer (7, 15, 16), patients receiving outpatient palliative care (9, 17), seriously ill older patients with complex medical conditions (18), or inpatients who received palliative care consultation after being admitted *via* an ED (3). By contrast, causes for admission of neurological inpatients receiving palliative care have not been studied so far. Here, we studied admission and palliative care needs in a sample of consecutive ED patients in a large university hospital.

## Methods

We studied 130 consecutive patients who had been admitted *via* the ED and subsequently received specialist palliative care consultations by a multi-professional palliative care service in the Department of Neurology, Charité—Universitätsmedizin Berlin, Campus Virchow-Klinikum, between January 2018 and December 2019. Ethical approval was given by the Ethics committee of the Charité—Universitätsmedizin Berlin (EA4/123/19).

Electronic medical records including ED documentation were retrospectively analyzed for age, gender, mode of admission, initial medical triage [Manchester Triage System, MTS (19)], level of consciousness [Glasgow Coma Scale, GCS, (20–22)], chief complaints, documentation of the (alleged) patient will concerning therapy limitations, (non)existence of a patient decree and healthcare proxy or legal guardian, medical imaging, initial treatment (medication), time to admission/time spent in the ED, admitting care units, time until specialist palliative care consultation was initiated, length of stay in the Department of Neurology, and mode of discharge. Time to admission was defined as the time of arrival at the ED to the time point of initial documentation of the receiving ward. Length of stay in the Department of Neurology was defined as the time from admission to the neurological ward to the day of discharge.

From palliative care assessment, routinely conducted by a multi-professional palliative care service at initiation of palliative care in all patients, we extracted the following variables: palliative care symptoms [Minimal Documentation System for Patients in Palliative Care, MIDOS, (23)], pain assessment (visual analog scale from 0 = no pain to 10 = worst pain possible), performance status [Eastern Cooperative Oncology Group, ECOG, (24)], and (non)existence of a patient decree, healthcare proxy, or legal guardian. If patients were not able to communicate, assessment was performed by relatives, proxy, palliative care service, or attending neurologist.

In addition, 14 neurological symptoms were systematically evaluated in reference to the MIDOS-rating scale by the attending neurologist, as they are not included in the routinely conducted palliative care assessment. We also added the items “diarrhea” and specified the item “dyspnea” through adding “dyspnea on resting” and “dyspnea on exertion” and the item “sleep disorder” through adding “difficulties to fall asleep” and “sleep disturbances” in the original MIDOS.

Descriptive statistics was performed *via* IBM SPSS (Statistical Package for the Social Sciences, IBM Corp., Version 23.0, Released 2014. Armonk, NY, USA). Metric data are presented as median (minimum–maximum). For data analysis of MIDOS and additional neurological symptoms, only answered items were included in analysis. Seven cases were excluded due to missing information.

## Results

One hundred thirty neurological inpatients (50% female, median age 69 years) who received palliative care after acute hospital admission were identified.

### Mode of Admission and Initial Medical Triage

Sixty-six percent of patients were brought to hospital by ambulance with or without an emergency physician, 11% came by patient transport ambulance, and 11% by other vehicles. For 12% of patients, means of transport was not documented. According to MTS, 45% were classified as requiring “immediate” or “very urgent” medical assessment. Forty-five percent were triaged as needing “urgent” or “standard” medical assessment. Eight percent were not triaged according to MTS, but instead labeled as “handed over from doctor to doctor” and tagged as “stroke” or “trauma.” In three cases, triage was not documented.

### Level of Consciousness and Chief Complaints on Admission

Level of consciousness was categorized as GCS 13–15 in 58% of patients. Eighteen percent were scored GCS 8–12 and 12% GCS 7 or below. In 12% of patients, a GCS score was not documented. Eight percent of patients required an invasive airway management (intubation or a supraglottic device).

**Figure 1:**
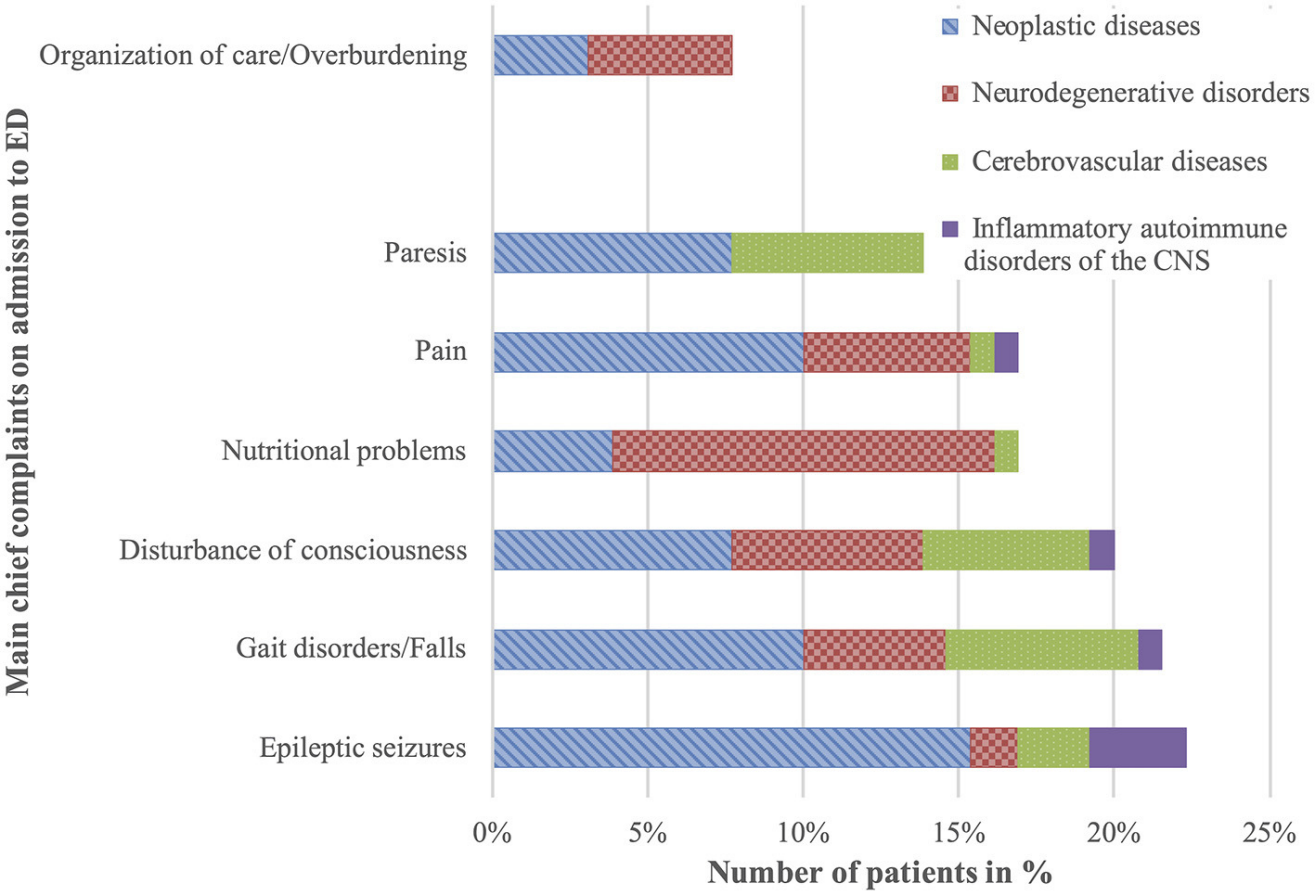
Main chief complaints on admission.

Altogether, 29 different chief complaints could be identified from emergency department documentation, with a median of 2 (1–5) complaints in each patient (Table 1). The most frequent reasons for acute admission were epileptic seizures (22%), gait disorder/falls (22%), disturbances of consciousness (20%), pain (17%), nutritional problems (17%), and paresis (14%). Difficulties with organization of care or overburdening of family were only mentioned in 8% (Figure 1).

**Table 1:** Frequent complaints on admission in a minimum of *n* ≥ 5 patients.

**Chief complaints on admission**	***n***	**%**
Epileptic seizures	29	22,3%
Gait disorder/falls	28	21,5%
Disturbance of consciousness	26	20,0%
Pain	22	16,9%
Nutritional problems/dysphagia	22	16,9%
Paresis	18	13,8%
Confusion	12	9,2%
Aphasia	11	8,5%
Organization of ambulant care/overburdening of family	10	7,7%
Infection	9	6,9%
Shortness of breath	7	5,4%
Nausea/vomiting	7	5,4%
Micturition disturbance	6	4,6%
Weakness	5	3,8%
Dizziness	5	3,8%
Paresthesia	5	3,8%

### Diagnostics and Therapy

The vast majority of patients received at least one mode of acute diagnostic imaging (88%). Cranial imaging was most frequently performed (60%). Fifty-eight percent received a cranial CT either without or with contrast medium (CM) and/or CT post-CM imaging; 2% had an initial cranial MRI. X-ray (29%) and CT (25%) of other body regions were frequently performed. Only 4% of patients were examined with ultrasound.

In 55% of cases, a medication was administered in the ED: antiseizure medication (19%) and benzodiazepines (14%), analgesics (WHO-I/antipyretics: 18%, WHO-II/III: 8%), antibiotics (17%), and anti-edematous treatment (17%) were initiated most commonly. Even 4% received systemic thrombolysis and/or recanalization as acute stroke treatment.

### Documentation of Healthcare Proxy or Legal Guardian, Patient Decree, and Therapy Limitations

In 31% of the ED documentations, we found statements concerning possible therapy limitations according to the (alleged) patient will, (non)existence of a patient decree, and (non)existence of a healthcare proxy or a legal guardian.

The existence of a healthcare proxy or legal guardian was documented in 15% of cases, whereas in 5% it was mentioned that there was none. The presence of a patient decree was documented in 8%; in 5%, it was explicitly noted that no patient decree exists.

The existence of therapy limitations was documented for 12% of patients. In 5%, it was explicitly stated that there are no limitations to therapy. In half of the cases with documented (non)existing therapy limitations, it was specified that the presumed wishes of the patient were considered with the help of family, healthcare proxy, or legal guardian. Two patients were directly quoted concerning their will. In one case, a conflict was mentioned between patient decree and the alleged patient will. In another case, it was documented that the alleged patient will still needs to be evaluated.

### Time to Admission, Admitting Care Units, and Diagnosis for Admission

Patients stayed 0.5–20 h (median 5 h) in the ED until they were admitted to a neurological ward (59%), intensive care unit (25%), or other departments (16%).

Primary diagnoses for admission were neoplastic disorders (49%), neurodegenerative disorders (30%), cerebrovascular diseases (18%), or inflammatory autoimmune disorders of the CNS (3%) (Table 2).

**Table 2:** Diagnoses.

**Diagnosis group**	**Diagnoses**	***n***	**%**
Neoplastic diseases	Primary brain tumors	22	16,9%
	Secondary brain tumors	30	23,1%
	Other neoplastic diseases	12	9,2%
Neurodegenerative disorders	Amyotrophic lateral sclerosis	17	13,1%
	Parkinson's disease	8	6,2%
	Atypical parkinsonism	4	3,1%
	Dementia	7	5,4%
	Other neurodegenerative disorders	3	2,3%
Cerebrovascular diseases	Ischemic stroke	15	11,5%
	Hemorrhagic stroke	8	6,2%
Inflammatory autoimmune disorders	Multiple sclerosis	4	3,1%

### Time to Initiation of Palliative Care, Palliative Care Needs, and Performance Status

In 63% of patients, it took at least 2 days until specialist palliative care consultation was initiated. In 25% of patients, it took 1 day. Only in 12% of patients were palliative care needs identified on admission.

**Figure 2:**
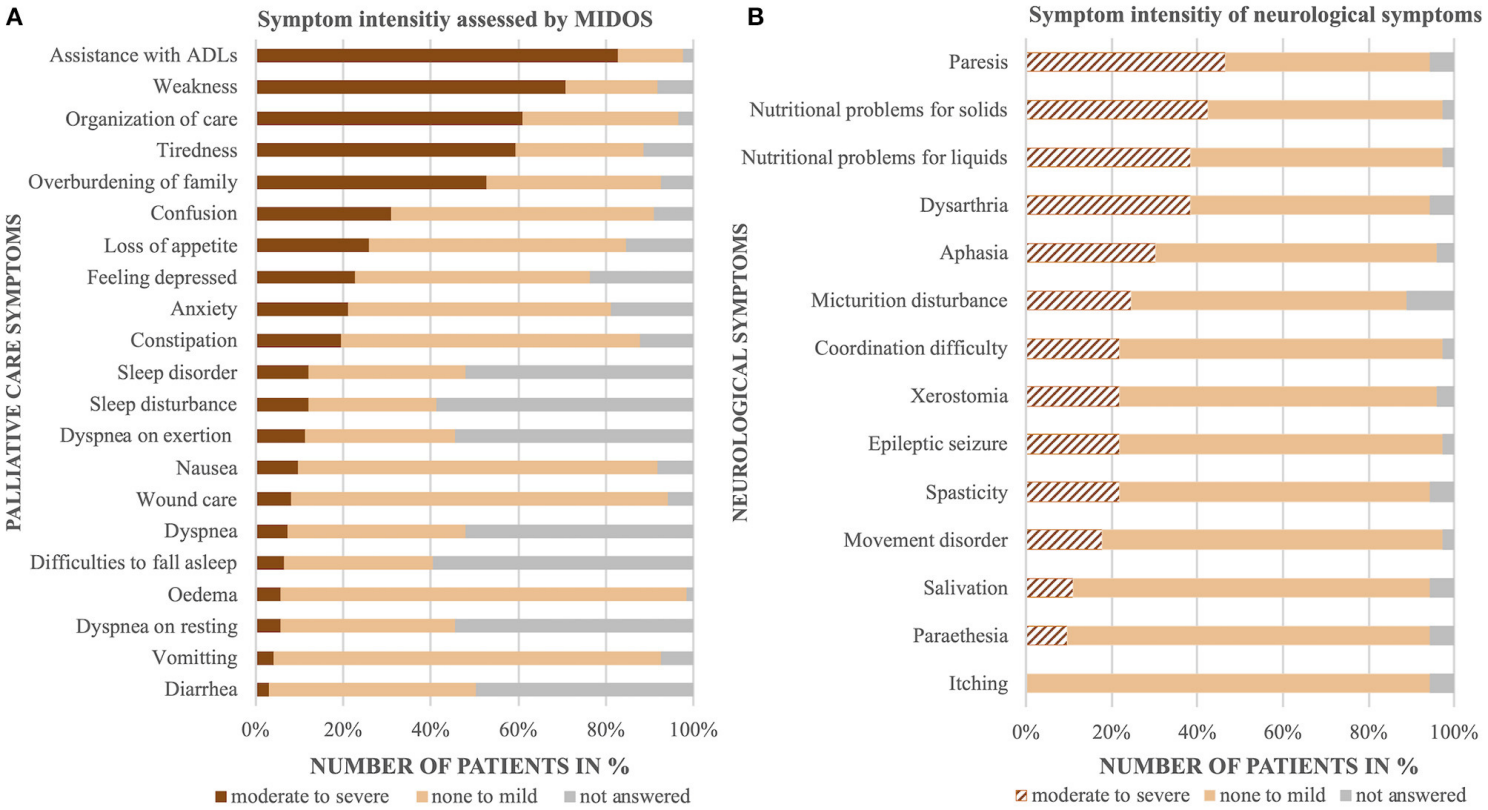
Palliative care assessment. Occurrence and intensity of palliative care symptoms **(A)** and complementary neurological symptoms **(B)**.

Symptom assessment after admission revealed that general symptoms and psychosocial problems such as assistance with Activities of Daily Living (ADL, 83%), weakness (71%), difficulties with organization of care (61%), tiredness (59%), or overburdening of family caregivers (53%) were key palliative care needs of at least moderate intensity (Figure 2A). Thirty-one percent of patients had experienced pain within the last 24 h, ranging from 3 to 10 points on the visual analog scale (4–6/10: 21%; 7–10/10: 10%), whereas 14% reported to have pain more than 3 during the assessment (4–6/10: 10%; 7–10/10: 4%). Complementary neurological symptoms were assessed in 73 patients. Difficulties in communication (30% aphasia, 38% dysarthria), nutritional problems for solids (42%) or fluids (38%), and paresis (47%) were the most common moderate to severe neurological symptoms (Figure 2B).

Performance status was highly impaired in most patients: 83% were “capable of only limited self-care” or “completely disabled” (ECOG 3 or 4), 17% were “restricted in physically strenuous activity” or “capable of all self-care but unable to carry out any work activities” (ECOG 1 or 2) (24).

### Length of Stay and Mode of Discharge

The median length of stay in the department of neurology was 10 days (2–44 days). Forty percent of patients were discharged with further inpatient (palliative care unit or hospice; 12%) or outpatient (home/nursery home with outpatient palliative care supply; 28%) specialized palliative care. Twenty-three percent were discharged without specialized palliative care supply. Twenty-two percent were transferred to other services (e.g., rehabilitation clinics). During their hospital stay, 15% of patients died; 47% while waiting for inpatient or outpatient palliative care.

## Discussion

Admission to hospital *via* the ED was triggered by acute events as well as exacerbation of potentially preventable or chronic medical problems.

The level of urgency assigned to the cause leading to admission may indirectly be indicated by means of transport to hospital as well as assessment on arrival. Most neurological patients with palliative care needs arrived by ambulance, a frequent mode of arrival of patients with palliative care needs (3, 9). However, mode of transport may also be influenced by frailty and high functional impairment, a barrier for self-organized arrival (2, 9). A straightforward indicator for acuteness of ED consultation is triage on arrival. More than half of our patients were assessed to be in need of urgent or immediate medical care or even of continuous monitoring. The proportion of patients needing prompt medical care thus seems slightly higher in neurological patients compared to other patients with palliative care needs presenting to the ED (3, 9). Consequently, it is not surprising that a relevant number of patients were initially admitted directly to the intensive care unit. An admission modality was not often reported in patients with other lifetime-reducing diseases and palliative care needs (7, 25). Frequent and elaborate diagnostics including neuroimaging and body imaging in the majority of patients as well as the variety of administered medications are not as easily conducted in an out-of-hospital setting. Admission to the ED therefore does not seem to be avoidable in all cases, as out-of-hospital treatment would not be equally available.

Patients presented to the ED with epileptic seizures, gait disorders, disturbances of consciousness, paresis, dysphagia, and nutritional problems. Less often, they also reported symptoms from the “classical” palliative spectrum such as pain, shortness of breath, nausea/vomiting, and weakness—symptoms already described in neurological inpatients receiving palliative care (1, 2, 26, 27). Compared to patients at large presenting to the ED, we unsurprisingly see a shift in frequency distribution of neurological chief complaints (28). This certainly reflects the most common diagnoses of neurological patients with palliative care needs: neoplastic diseases (i.e., primary and secondary brain tumors or meningeal carcinomatosis), neurodegenerative disorders, and cerebrovascular diseases.

Chief complaints and symptoms are frequently ambiguous and may be caused both by true neurological emergencies and by persistent deficits that do not require in-hospital treatment. Paresis, epileptic seizure, and disturbance of consciousness may be seen as chief complaints that may require urgent diagnostics and possibly treatment. Epileptic seizures for example are common in older adults as well as cancer patients, and their occurrence is associated with a significant morbidity and mortality (16, 29, 30). First occurrence of epileptic seizures or status epilepticus should lead to neuroimaging and diagnostics. Also, in recurring seizures, reimaging may be needed to exclude tumor progression or complications in primary or secondary brain tumor patients. Although our data do not gather information whether the event was new or reoccurring, seizures may be considered as events that lead to almost unavoidable admission, especially seizures with impaired awareness like tonic–clonic seizures. Even in patients already receiving outpatient palliative care, neurological complications usually require acute hospitalization (25). For example, in patients with sudden onset of paresis due to an ischemic stroke, an immediate ED admission enables treatment options (thrombolysis and/or recanalization) with a chance to prevent major disability, also in palliative care patients.

Other chief complaints like pain, nutritional problems, and gait disorders or falls can be argued to be problems that could also be sufficiently dealt with in an out-of-hospital setting and may therefore be potentially preventable causes for admission. Nevertheless, dysphagia and nutritional problems are still the most frequent symptoms in hospitalized patients with amyotrophic lateral sclerosis (4). In Parkinson's disease, complications due to falls or reduced ingestion remain common causes for emergency department admissions and hospitalization (31).

A precise documentation of the palliative situation as well as a clearly documented will concerning treatment limitations may help to avoid stressful diagnostics and treatment as well as unnecessary hospitalization. End-of-life discussions also have the potential to reduce the risk of more than one ED visit before death (15). However, in ED documentation information about advance care planning, the (alleged) patient will or healthcare proxy remains sparse (3, 5). This is remarkable, as decisions made in the emergency department often affect the trajectory of in-hospital care. Certainly obstacles such as urgency of the medical situation, lack of an adequate private and calm setting, and lack of knowledge of the complete medical history of the patient are a challenge for healthcare providers, patients and their families (32). However, discussion of goals of care and life-sustaining treatment is essential to conducting treatment in alignment with the patient's will. In neurology and beyond, malignant stroke and massive intracranial hemorrhage are well-acknowledged acute events that initiate serious illness conversations. However, there are also other, disease-specific well-defined “triggers” (33). In neurodegenerative diseases, for example dysphagia and associated nutritional problems are seen as such event-driven milestones to initiate serious illness conversation (33).

Neurological inpatients are known to have specific palliative care needs (1, 2, 27). Our data show that emergency assessment only reveals a small fraction of the full multidimensionality of symptom burden. Reasons for admission display known categories for palliative care patients with other lifetime-reducing diseases: exacerbation of known or occurrence of new symptoms, worsening performance status, and disturbances of consciousness (3, 6, 15, 18). Psychosocial problems like organization of ambulant care or overburdening of family caregivers were rarely obvious as an initial cause of admission. Rather, they became evident during the in-hospital stay in the majority of inpatients and are key palliative care needs of at least moderate intensity. On the other hand, chief complaints presented in the ED like disease-specific problems affecting mobility, nutrition, and communication were consistent with the most common moderate to severe neurological symptoms evaluated by palliative care assessment. Epileptic seizures were frequent chief complaints on admission, but less frequently mentioned as major problems in palliative care assessment later on. A possible explanation might be an already successful establishment or optimization of antiseizure medication.

In the majority of our patients, it took more than 2 days to initiate palliative care. A considerable number of patients were able to be transferred to a palliative care unit, hospice, or outpatient specialized palliative care providers. However, almost half of the patients who died during their hospital stay were waiting for such a transfer. Early identification of palliative care needs and an early decision-making concerning mode of discharge may be important to enable a transfer to further specialized palliative care supply.

Taken together, our results suggest the need for adaption, further validation, and use of a screening tool that could help to increase awareness of unmet palliative care needs of neurological patients in the ED (32–34). The variables we propose to incorporate in such a screening tool would be symptom burden, functional status, and estimated prognosis. Trigger for ED clinicians to apply such screening tool should be a diagnosed or highly suspected, life-limiting primary neurological disease or affection of the nervous system by other life-limiting illnesses. For the variable “symptom burden,” we would suggest that the existence of a minimum of two uncontrolled (neurological and/or palliative care) symptoms of at least two different dimensions (physical, social, psychological) should be required, as our patients presented with a median of two chief complaints. For assessment of the variable “functional status,” we would suggest using ECOG as a straightforward and well-established tool in palliative care assessment, which has also shown to be associated with prognosis (24, 34, 35). For prognosis estimation, we would incorporate the 12-month “surprise” question (12-SQ), as it has shown to help in assessing the urgency of palliative care integration in oncological as well as neurological patients (34–37).

We believe that such an instrument could help to initiate specialized palliative care consultation as early as possible in the trajectory of in-hospital care.

## Limitations

Firstly, our study focused on patients who were admitted *via* the ED and consequently received palliative care in a neurological ward. Those who were initially admitted solely for end-of-life care are not systematically included, because they do not regularly receive specialized palliative care through our consultation service. In addition, no standardized assessment was performed to decide whether a patient should receive palliative care consultation or not. Patients who were able to be discharged from ED were also not considered in this study. Therefore, we cannot quantify the overall number of patients with neurological chief complaints and palliative care needs who present to the ED.

Secondly, our study is restricted by its retrospective design. ED documentation usually is a brief summary of patients' complaints. Missing information can be either because information was not gathered or because documentation was failed. It also leaves questions like how many patients were already receiving palliative care before being admitted to the ED unanswered—an aspect that definitively should be considered in future, prospective studies.

## Conclusion

Causes for admission of neurological patients with palliative care needs are broad and include acute events, exacerbation of chronic symptoms, and potentially avoidable problems. Patients already present with complex symptom burden in the ED. However, ED assessment is not sufficient to display the full multidimensionality especially when it comes to psychosocial problems. Prospective studies should follow to develop short screening tools to identify palliative care needs of patients with chronic neurological diseases at the very beginning: in the emergency department.

## Data Availability Statement

The raw data supporting the conclusions of this article will be made available by the authors, without undue reservation.

## Ethics Statement

The studies involving human participants were reviewed and approved by The Ethics Committee of Charité—Universitätsmedizin Berlin. Written informed consent for participation was not required for this study in accordance with the national legislation and the institutional requirements.

## Author Contributions

A-CW designed the study, acquired the data, performed the descriptive statistics, and drafted the manuscript. CP provided important methodological advice and revised the manuscript. AK designed the study, acquired the data, supervised the study, and revised the manuscript for intellectual content. All authors contributed to the article and approved the submitted version.

## Conflict of Interest

The authors declare that the research was conducted in the absence of any commercial or financial relationships that could be construed as a potential conflict of interest.

## Publisher's Note

All claims expressed in this article are solely those of the authors and do not necessarily represent those of their affiliated organizations, or those of the publisher, the editors and the reviewers. Any product that may be evaluated in this article, or claim that may be made by its manufacturer, is not guaranteed or endorsed by the publisher.
